# Functional independence of Taiwanese patients with mucopolysaccharidoses

**DOI:** 10.1002/mgg3.790

**Published:** 2019-06-18

**Authors:** Chung‐Lin Lee, Hsiang‐Yu Lin, Chih‐Kuang Chuang, Huei‐Ching Chiu, Ru‐Yi Tu, You‐Hsin Huang, Wuh‐Liang Hwu, Fuu‐Jen Tsai, Pao‐Chin Chiu, Dau‐Ming Niu, Yann‐Jang Chen, Mei‐Chyn Chao, Tung‐Ming Chang, Ju‐Li Lin, Chia‐Ying Chang, Yu‐Chia Kao, Shuan‐Pei Lin

**Affiliations:** ^1^ Department of Pediatrics Mackay Memorial Hospital Hsinchu Taiwan; ^2^ Department of Pediatrics Mackay Memorial Hospital Taipei Taiwan; ^3^ Department of Medicine Mackay Medical College New Taipei City Taiwan; ^4^ Division of Genetics and Metabolism, Department of Medical Research MacKay Memorial Hospital Taipei Taiwan; ^5^ Mackay Junior College of Medicine, Nursing and Management Taipei Taiwan; ^6^ Department of Medical Research China Medical University Hospital, China Medical University Taichung Taiwan; ^7^ College of Medicine Fu‐Jen Catholic University Taipei Taiwan; ^8^ Department of Pediatrics National Taiwan University Hospital Taipei Taiwan; ^9^ Department of Medical Research, Genetics Center China Medical University Hospital Taichung Taiwan; ^10^ Department of Pediatrics Kaohsiung Veterans General Hospital Kaohsiung Taiwan; ^11^ Institute of Clinical Medicine National Yang‐Ming University Taipei Taiwan; ^12^ Department of Pediatrics Taipei Veterans General Hospital Taipei Taiwan; ^13^ Department of Pediatrics, Renai Branch Taipei City Hospital Taipei Taiwan; ^14^ Department of Pediatrics Kaohsiung Medical University Hospital Kaohsiung Taiwan; ^15^ Department of Pediatric Neurology Changhua Christian Children's Hospital Changhua Taiwan; ^16^ Department of Biological Science and Technology, College of Biological Science and Technology National Chiao Tung University Hsinchu Taiwan; ^17^ Department of Pediatrics Chang‐Gung Memorial Hospital Taoyuan Taiwan; ^18^ Department of Pediatrics E‐DA Hospital Kaohsiung Taiwan; ^19^ Department of Infant and Child Care National Taipei University of Nursing and Health Sciences Taipei Taiwan

**Keywords:** independent living, mucopolysaccharidosis, Taiwan, WeeFIM

## Abstract

**Background:**

Information on functional strengths and weaknesses of mucopolysaccharidosis (MPS) patients is important for early intervention programs and enzyme replacement therapy (ERT).

**Methods:**

We used the Functional Independence Measure for Children (WeeFIM) questionnaire to assess the functional skills of 63 Taiwanese MPS patients (median age, 13 years 3 months; range, 3–20 years) from January 2012 to December 2018.

**Results:**

Mean total WeeFIM score was 75.4 of a potential score of 126. Mean total WeeFIM scores of each type (MPS I, MPS II, MPS IIIB, MPS IVA, and MPS VI) were 103.8, 76.2, 41.6, 92.2, and 113.6, respectively. Mean scores for self‐care, mobility, and cognition domains were 30 (maximum 56), 23 (maximum 35), and 22 (maximum 35), respectively. MPS type IIIB patients had the lowest scores in self‐care, mobility, cognition, and total domains compared to other types of MPS. All patients with ERT in MPS I, II, and IVA had higher scores in self‐care and mobility domains than patients without ERT. Most patients required assistance for self‐care skills, especially in grooming and bathing.

**Conclusion:**

MPS patients require support and supervision in self‐care tasks. For cognition tasks, MPS IIIB patients also require help. This questionnaire is useful to identify the strengths and limitations of MPS patients.

## 1 INTRODUCTION

The mucopolysaccharidoses (MPSs) are a group of lysosomal storage disorders that are deficient in enzymes catalyzing the degradation of glycosaminoglycans. Accumulation of glycosaminoglycan molecules results in cell, tissue, and organ dysfunction. Eleven known enzyme deficiencies cause seven distinct MPS types (I, II, III, IV, VI, VII, and IX), and are inherited in an autosomal recessive manner except for MPS II (Hunter syndrome), which is transmitted as an X‐linked recessive disorder (Chuang & Lin, 2007; Neufeld & Muenzer, 2001). Each MPS type shows a wide spectrum of clinical severity. Severe and attenuated forms exist for MPS I (Hurler, Hurler–Scheie and Scheie syndromes) and MPS II (Hunter syndrome), and additional subtypes have been described for MPS III and IV. It is worth noting that severity in MPS I and II is defined by the involvement of CNS and the presence of cognitive impairment. The incidence of MPS is reported to be 1.9–4.5/100,000 live births (Lin et al., 2009). In Taiwan and other Asian countries, the most common type is MPS II, compared to MPS I or MPS III in most Caucasian countries.

Parents of children with a congenital anomaly want to know how well the child will function in life. It is very important for pediatricians to provide accurate information to these families. In addition, the information is needed to plan educational, vocational, long‐term care, and accommodation programs and services. The Functional Independence Measure for Children (WeeFIM) questionnaire is a convenient tool to assess functional skills, and has been modified for use in Chinese children (Wong, Wong, Chan, & Wong, 2002). We used the WeeFIM to assess functional independence in Taiwanese children 2–20 years old with MPS and provide a reference for family support and educational services in early intervention programs.

## 2 METHODS

### 2.1 Study population

A total of 63 children between 2 and 20 years old with MPS and their parents were recruited at seven medical centers in Taiwan (Mackay Memorial Hospital, Taipei Veterans General Hospital, Kaohsiung Veterans General Hospital, China Medical University Hospital, National Taiwan University Hospital, National Cheng Kung University Hospital, and Changhua Christian Children's Hospital) from January 2012 to December 2018. The parents and children completed the WeeFIM questionnaire at the clinic. The ethics committee of the hospitals approved the study protocol and all participants or their parents provided written informed consent.

### 2.2 WeeFIM questionnaire

Wong et al. (2002) translated the content of the WeeFIM questionnaire into Chinese and used it to estimate functional independence in children 6 months to 7 years old. It has been used to evaluate children with developmental disabilities who were up to 21 years old. This questionnaire is designed for primary caregivers (parents or teachers) who know the child well to observe their child's abilities directly. Advantages of the WeeFIM are its conciseness (simple scoring of 1–7), comprehensiveness (covering all developmental aspects), shorter administration time (it can be administered in ≤20 min), and the discipline‐free requirements (Wong et al., 2002). The questionnaire includes 18 items in three domains: self‐care including eating, grooming, bathing, dressing upper, dressing lower, toileting, bladder, and bowel, mobility including chair transfer, toilet transfer, tub transfer, walking, and stairs, and cognition including comprehension, expression, social interaction, problem‐solving, and memory. The responses are scored as: 1, total assistance required; 2, maximal contact assistance or prompting with 25%–49% performance effort; 3, moderate contact assistance or prompting with 50%–74% performance effort; 4, minimal contact assistance or prompting with >75% performance effort; 5, supervision, setup, or standby prompting; 6, modified independence using an assistive device, or not safe or timely performance; and 7, complete independence without a helper or device, safe, and timely performance (Table 1) (Sperle, Ottenbacher, Braun, Lane, & Nochajski, 1997). Scores from 1–5 indicate that the child is dependent and needs help in performing daily activities, while 6–7 indicate independence with no help required. The self‐care, mobility, and cognition domain scores ranged from 8–56, 5–35, and 5–35, respectively. The total score ranged from 18–126 (Lin et al., 2012).

**Table 1:** Functional independence measure (WeeFIM) for children

Levels	
Independent	
7. Complete independence (timely, safely)	No
6. Modified independence (device)	Helper
Modified dependence	
5. Supervision	Helper
4. Minimal assistance (subject = 75% +)	
3. Moderate assistance (subject = 50% +)	
Complete dependence	
2. Maximal assistance (subject = 25% +)	
1. Total assistance (subject = 0% +)	
Items	Grade
Self‐care	
1. Eating (Ea)	
2. Grooming (Gr)	
3. Bathing (Ba)	
4. Dressing upper (DU)	
5. Dressing lower (DL)	
6. Toileting (To)	
7. Bladder (Bl)	
8. Bowel (Bo)	
Mobility	
9. Bed/Chair/Wheelchair transfer (ChT)	
10. Toilet transfer (ToT)	
11. Tub/Shower transfer fTuT)	
12. Walk/Wheelchair (WC)	
13. Stairs (St)	
Cognition	
14. Comprehension (Co)	
15. Expression (Ex)	
16. Social interaction (S!)	
17. Problem‐solving (PS)	
18. Memory (Me)	

### 2.3 Statistical analysis

The 63 children were stratified into groups of 0–6, 6–12, 12–18, and 18–20 years old for evaluation of functional performance. Linear regression analysis was used to compare the WeeFIM scores of the four age groups. Pearson's correlation coefficient (r) was used to determine the relationships of age and the 18 WeeFIM subscores, and significance was tested by Fisher's r to z transformation. The WeeFIM scores were analyzed with age as a continuous variable, and sex (male or female) and MPS subtype as class variables using 2‐way analysis of variance. SPSS version 11.5 (SPSS, Inc., Chicago, IL, USA) was used to perform the statistical analysis. Statistical significance was set at *p* < 0.05.

## 3 RESULTS

**Figure 1:**
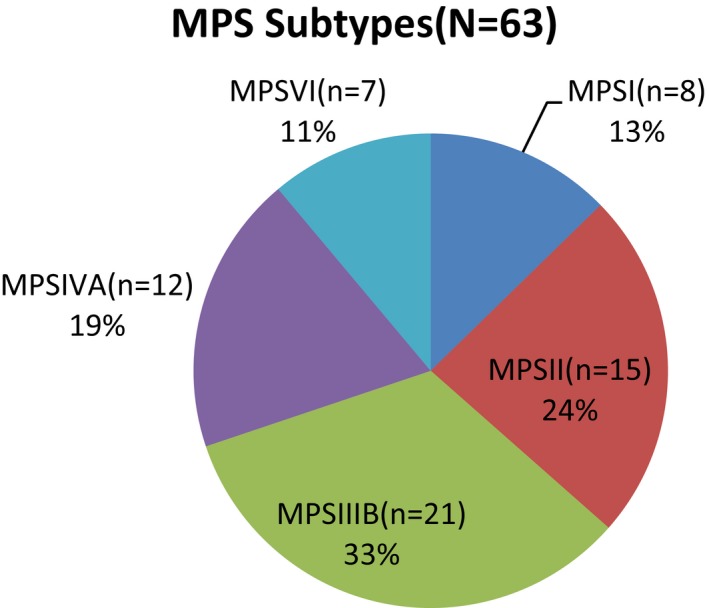
The subtype of MPS patients. MPS, mucopolysaccharidosis

**Figure 2:**
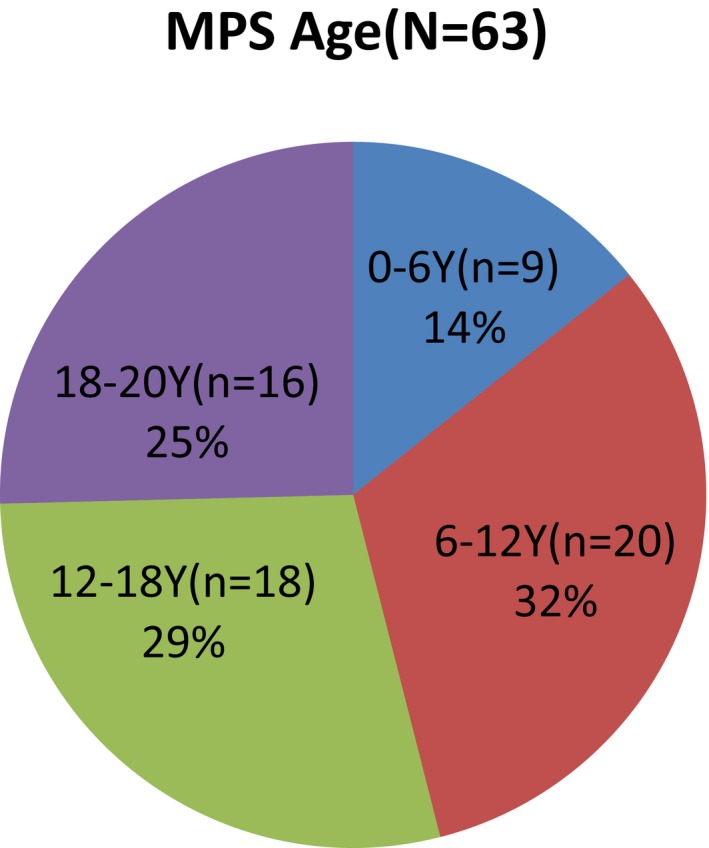
The age of MPS patients. MPS, mucopolysaccharidosis

Of the 63 children enrolled in this study, 37 were boys and 26 were girls, and 9, 20, 18, and 16 were 0–6, 6–12, 12–18, and 18–20 years old, respectively (median age 13 years 3 months) and eight patients had MPS I, while 15, 21, 12 and seven had MPS II, IIIB, IVA, and VI, respectively (Figures 1 and 2). WeeFIM total scores ranged from 39 to 126 (mean, 103.8), with a mean quotient of 85% compared to normative data for Chinese children (Wong et al., 2004). Table 2 summarizes the total, mean, and median scores; mean quotients; and interquartile ranges for each domain in the five subtype groups of MPS. Mean self‐care, mobility, and cognition domain scores were 30 (maximum 56, mean quotient 56%), 23 (maximum 35, mean quotient 66%), and 22 (maximum 35, mean quotient 65%), respectively.

**Table 2:** WeeFIM scores among children with mucopolysaccharidosis

Group	MPSI	MPSII	MPSIIIB	MPSIVA	MPSVI	Total
*n*	8	15	21	12	7	63
Self‐care score						
Range	8–56	8–56	8–31	11–56	36–55	8–56
Mean score	38	35	14	36	47	30
Median score	21	37	11	35	47	25
Interquartile range	31	42	12	31	3	39
Mobility score						
Range	5–35	5–35	5–35	10–35	25–35	5–35
Mean score	24	26	17	24	32	23
Median score	29	32	17	25	33	25
Interquartile range	20	15	17	12	2	21
Cognition score						
Range	30–35	5–35	5–32	19–35	33–35	5–35
Mean score	34	10	10.	32	34	22
Median score	35	8	8	35	35	28
Interquartile range	0	8	8	4	2	27
Total score						
Range	48–126	18–126	18–98	48–126	104–123	18–126
Mean score	97	81	42	92	114	75
Median score	107	87	34	97	115	69
Interquartile range	52	75	40	49	10	76

Note

a. Comparisons to normative data for Chinese children. Maximum possible scores: total 126; self‐care 56; motility 35; cognition 35.

Abbreviation: MPS, mucopolysaccharidosis.

**Figure 3:**
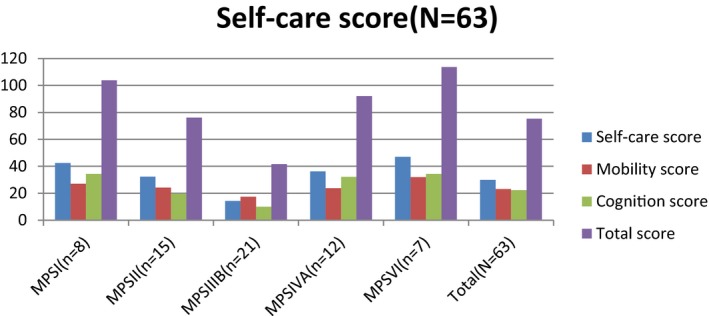
Self‐care, mobility, cognition, and total score in MPS patients. MPS, mucopolysaccharidosis

Figure 3 showed the scores for different types of MPS in self‐care, mobility, cognition, and total domains. MPS IIIB patients had the lowest scores in self‐care, mobility, cognition, and total domains compared to other types of MPS. WeeFIM total score and 18 subscores for the three domains were positively correlated with age (*p* < 0.05, Figure 4). MPS I and MPS II patients had positively correlation with age in cognition domain (Figures 4‐1 and 4‐2). Because MPS IIIB patients had most severe cognitive impairment, they did not have positively correlation with age in cognition domain (Figure 4‐3). MPS IVA and MPS VI patients had no correlation between age in cognition domain (Figures 4‐4 and 4‐5) due to no cognitive impairment. The best performance was in the mobility domain (mean quotient, 66%) and the worst was in the self‐care domain (mean quotient, 56%). Most of these patients required assistance for the self‐care skills, especially in grooming and bathing.

**Figure 4:**
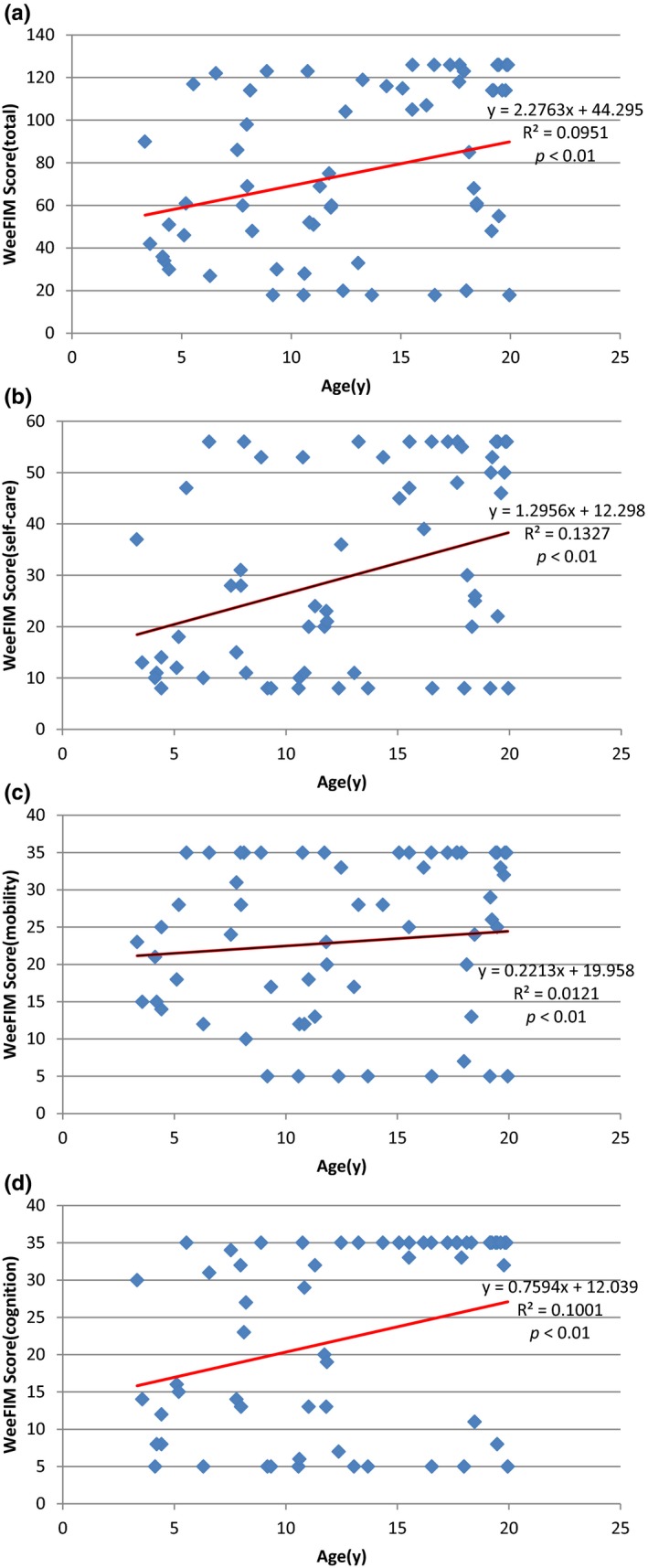
Correlation of age and (a) Total WeeFIM score and (b) Self‐care domain, (c) Mobility domain, and (d) Cognition domain scores in this series of Taiwanese children with mucopolysaccharidoses (*n* = 63). All four scores increased progressively with age (*p* < 0.01)

**Figure 4‐1:**
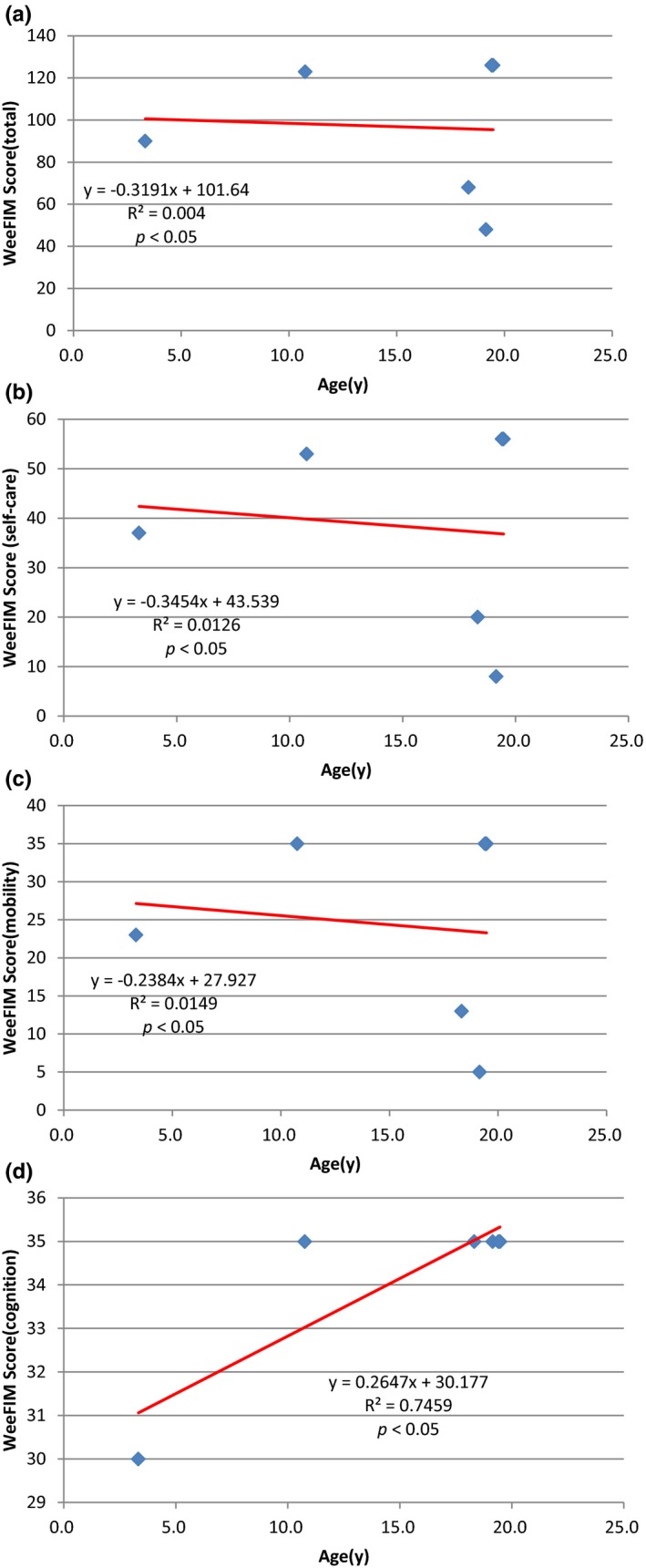
(MPS I). Correlation of age and (a) Total WeeFIM score and (b) Self‐care domain, (c) Mobility domain, and (d) Cognition domain scores in this series of Taiwanese children with mucopolysaccharidoses (*n* = 8). Cognition domain scores increased progressively with age (*p* < 0.05). MPS, mucopolysaccharidosis

**Figure 4‐2:**
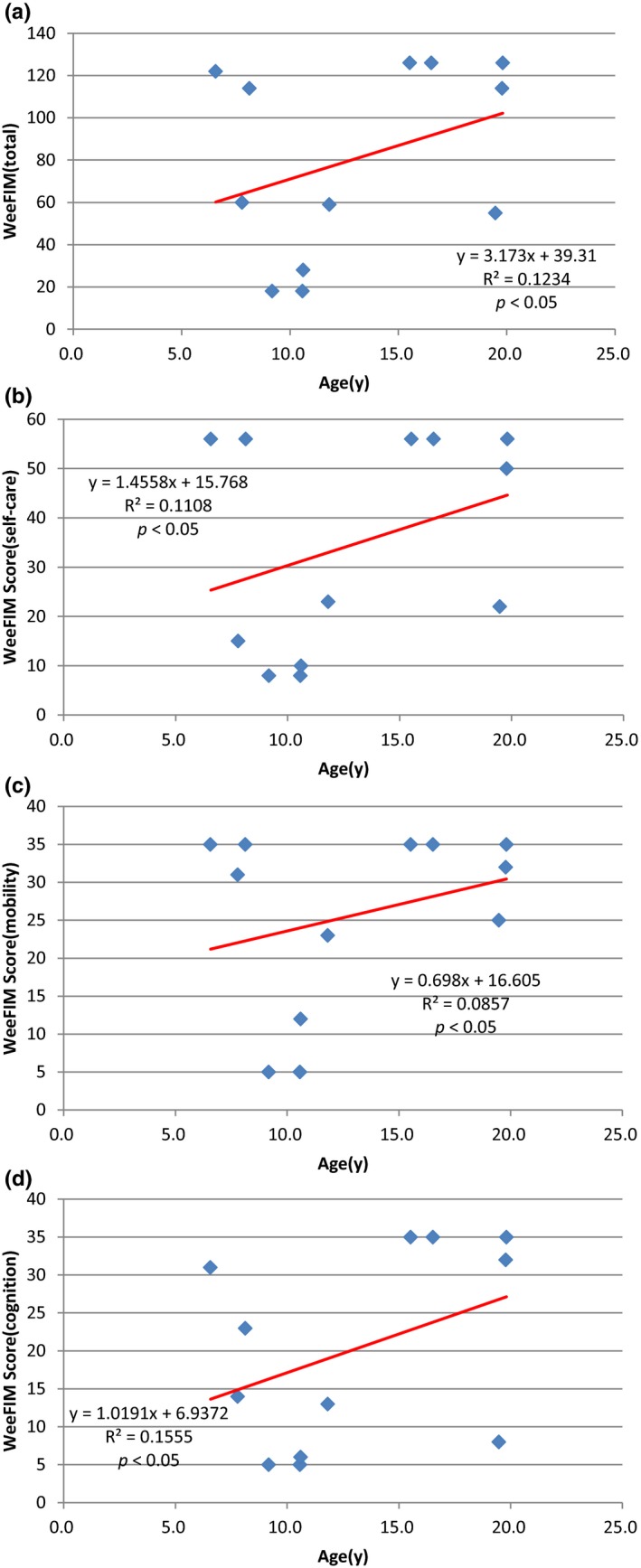
(MPS II). Correlation of age and (a) Total WeeFIM score and (b) Self‐care domain, (c) Mobility domain, and (d) Cognition domain scores in this series of Taiwanese children with mucopolysaccharidoses (*n* = 15). All four scores increased progressively with age (*p* < 0.05). MPS, mucopolysaccharidosis

**Figure 4‐3:**
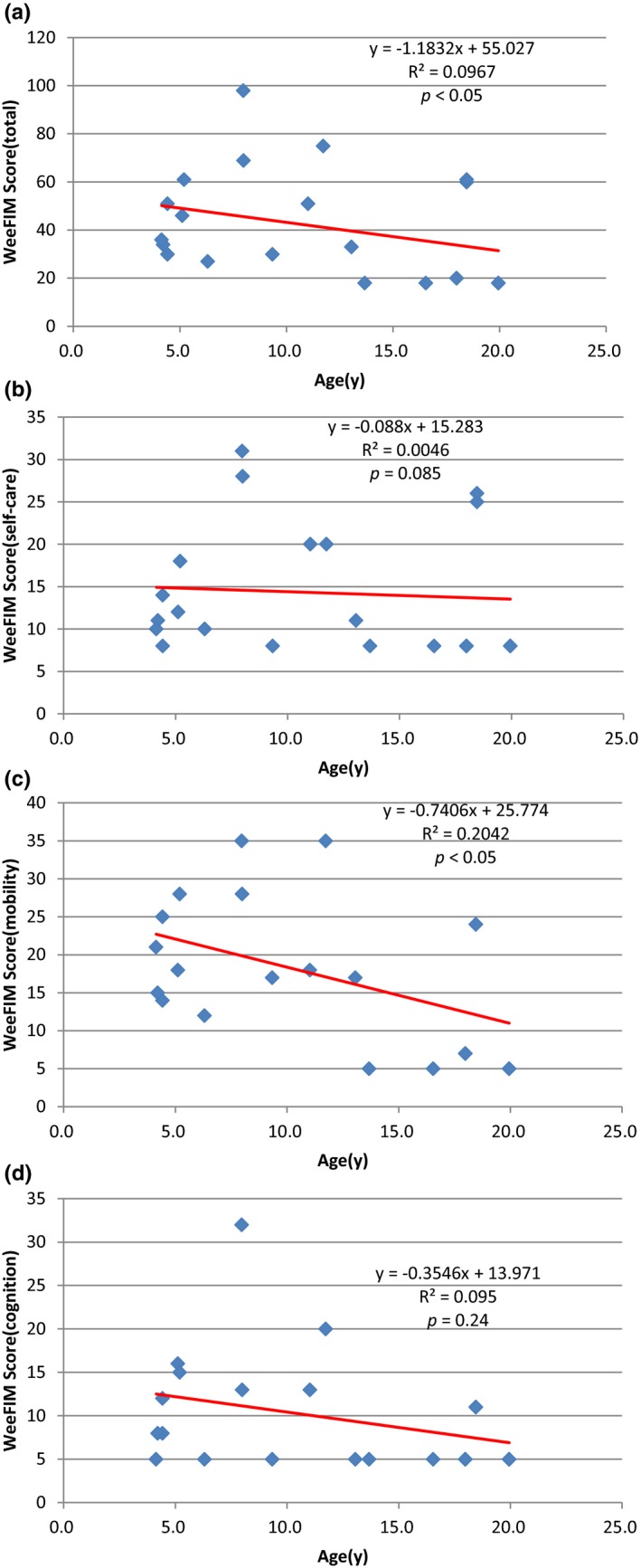
(MPS IIIB). Correlation of age and (a) Total WeeFIM score and (b) Self‐care domain, (c) Mobility domain, and (d) Cognition domain scores in this series of Taiwanese children with mucopolysaccharidoses (*n* = 21). Cognition domain scores did not increase progressively with age (*p* = 0.24). MPS, mucopolysaccharidosis

**Figure 4‐4:**
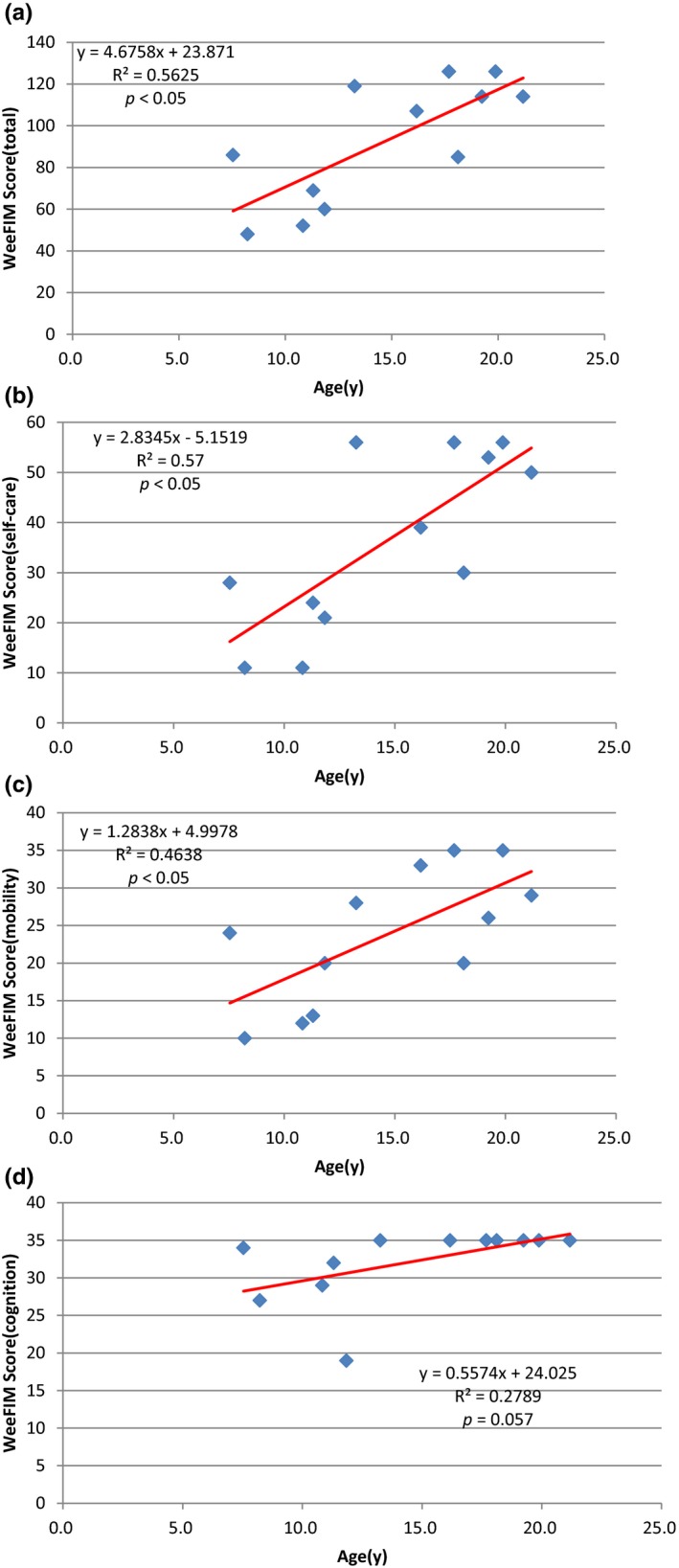
(MPS IVA). Correlation of age and (a) Total WeeFIM score and (b) Self‐care domain, (c) Mobility domain, and (d) Cognition domain scores in this series of Taiwanese children with mucopolysaccharidoses (*n* = 12). Cognition domain scores did not increase progressively with age (*p* = 0.057). MPS, mucopolysaccharidosis

**Figure 4‐5:**
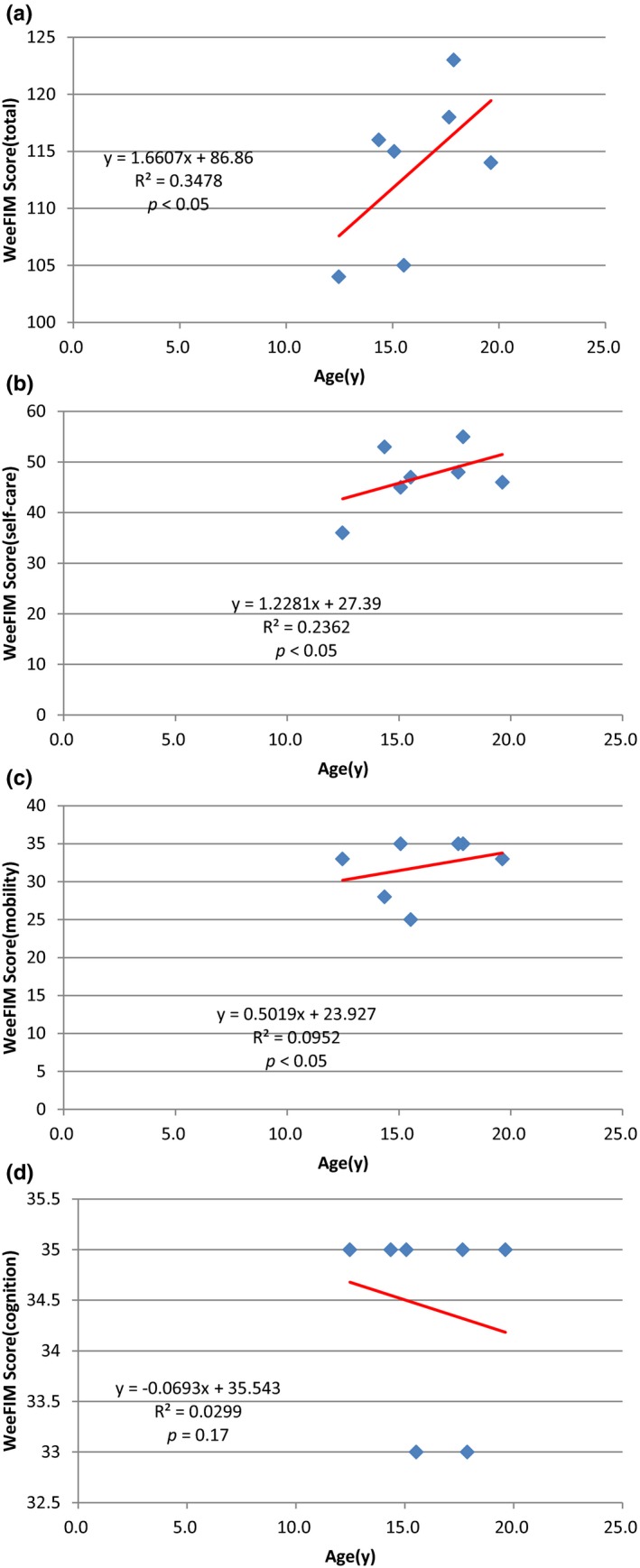
(MPS VI). Correlation of age and (a) Total WeeFIM score and (b) Self‐care domain, (c) Mobility domain, and (d) Cognition domain scores in this series of Taiwanese children with mucopolysaccharidoses (*n* = 7). Cognition domain scores did not increase progressively with age (*p* = 0.17). MPS, mucopolysaccharidosis

## 4 DISCUSSION

**Figure 5:**
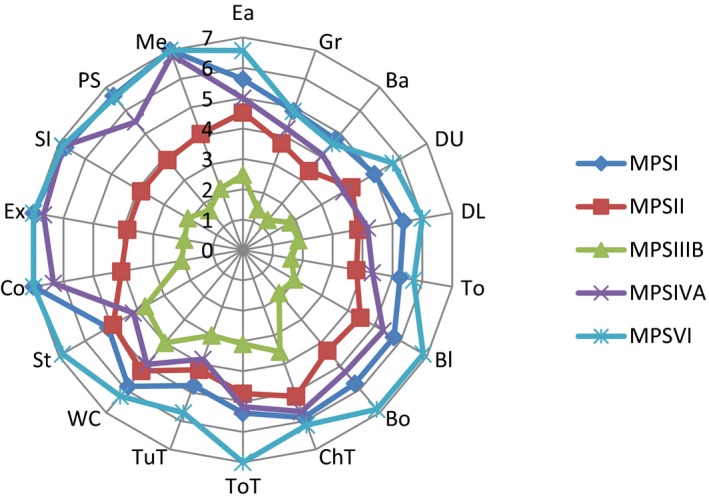
WeeFIM profiles of the study participants stratified by subtype of MPS. MPS I (n=8); MPS II (n=15); MPS IIIB (n=21); MPS IVA (n=12); MPS VI (n=7). Ba=bathing; Bl=bladder; Bo=bowel; ChT=bed/chair/wheelchair transfer; Co=comprehension; DL=dressing (lower); DU=dressing (upper); Ea=eating; Ex=expression; Gr=grooming; Me=memory; PS=problem‐solving; SI=social interaction; St=stairs; To=toileting; ToT=toilet transfer; TuT=tub/shower transfer; WC=walk/wheelchair

The WeeFIM questionnaire was used to analyze delays in cognition, self‐care, and mobility, and to provide data on the range of functional performance across these three domains in a group of children with MPS 3 years 3 months to 20 years old. According to the WeeFIM profiles of the study participants stratified by subtype of MPS (Figure 5), we found that the strongest performance was in the mobility domain and the weakest was in the self‐care domain. MPS IIIB patients had the lowest scores in self‐care, mobility, cognition, and total domains compared to other types of MPS, as reported previously (Buhrman, Thakkar, Poe, & Escolar, 2014; Delgadillo, O'Callaghan, Gort, Coll, & Pineda, 2013; Héron et al., 2011; Jansen et al., 2007; Lin et al., 2018; Malm & Månsson, 2010; Meyer et al., 2007; Ruijter et al., 2008; Truxal et al., 2016; Valstar et al., 2010; Velasco, Sanchez, Martin, & Umaña, 2017). MPS II patients had poor abilities in cognition domain because this study included severe type MPS II patients (Wraith et al., 2008). Most of these patients required assistance with self‐care skills, especially in grooming and bathing, because glycosaminoglycan molecules accumulate in tissue to restrict joint range of motion.

**Figure 6:**
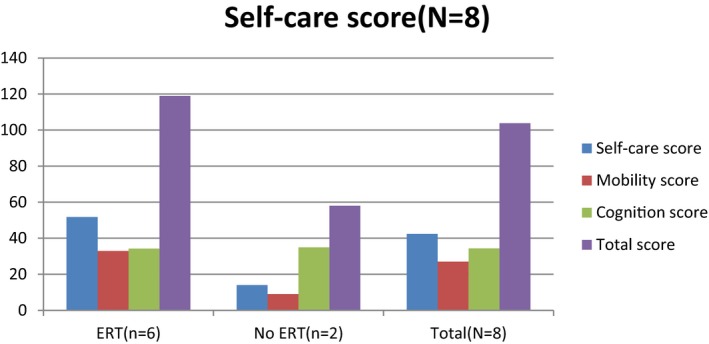
WeeFIM scores in MPS I patients between ERT and non‐ERT

**Figure 7:**
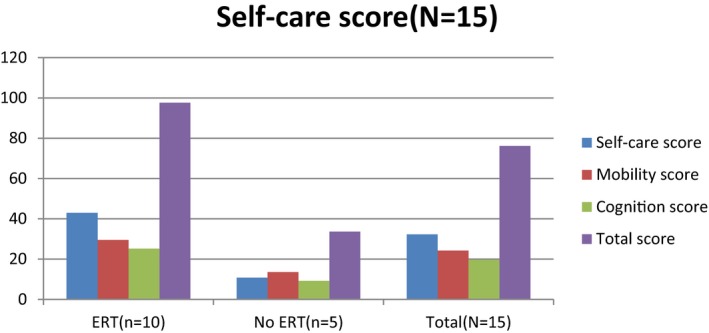
WeeFIM scores in MPS II patients between ERT and non‐ERT

**Figure 8:**
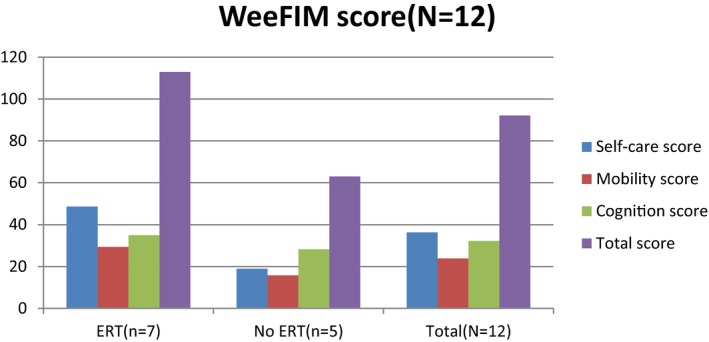
WeeFIM scores in MPS IVA patients between ERT and non‐ERT

**Figure 9:**
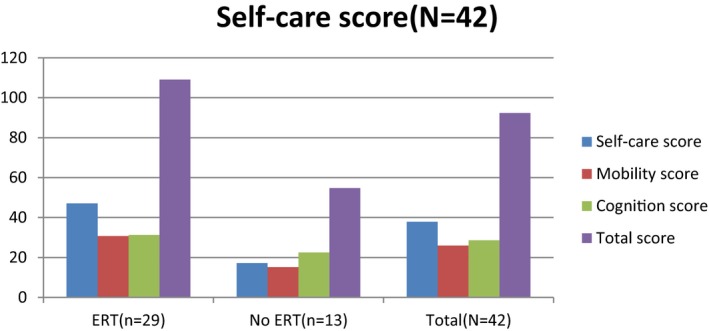
WeeFIM scores in all MPS patients between ERT and non‐ERT

Enzyme replacement therapy (ERT) is an effective treatment for MPS patients to reduce the accumulation of glycosaminoglycans and improve the restriction of joint range (Hendriksz et al., 2016). We compared the WeeFIM score in all the MPS patients (including those with MPS I, II, and IVA and total patients; MPS VI patients were not included because only one patient had ERT) between ERT and non‐ERT (Figures 6, 7, 8, 9). Patients with ERT (*n* = 29) and MPS I, II, and IVA had higher scores in self‐care and mobility domains than patients without ERT did (*p* < 0.05). MPS II and IVA patients with ERT had better performance in cognition domain than patients without ERT did (*p* < 0.05). MPS I patients with ERT had no apparent improvement than those without ERT (*p* = 0.271). All of our MPS I patients classified with Scheie syndrome (mild MPS I form) had cognitive abilities that were as normal as those of healthy people. Among all the types of MPS patients, we found that patients with ERT had better performance in every domain than patients without ERT did. However, it was significant that we would not start ERT in patients with a severe disease due to irreversible CNS and cognitive impairment. Patients who could start ERT had relatively slight symptoms and made considerable improvement after ERT.

The WeeFIM is a reliable instrument to evaluate the functional status of children with disabilities. It was first validated in American children (Msall et al., 1994) and, then, was used by Wong et al. and others (Ottenbacher et al., 1999; Wong et al., 2002) in similar studies. In a study conducted in Hong Kong, the Chinese‐language version of the WeeFIM that was developed by Wong et al. (2002) obtained results consistent with earlier reports in Japanese children (Wong & Wong, 2007). The WeeFIM has been proven in different cultures and languages to be a good tool to assess the strengths and weaknesses of participants with disabilities; however, it does not replace other psychological tests that evaluate adaptation, intelligence, or communication.

Children with MPS have relatively poor self‐care skills. These results help in designing early interventions to foster self‐care and independent living (Lin et al., 2016). Occupational therapy, physical and sensory integration, special education, and improving social behavior depend on psychotherapists, physiotherapists, occupational therapists, pediatricians, and parents. The effectiveness of such programs would be improved by the intervention of concerned parents with simple daily training. Because children with MPS usually start school at the same age as their peers, the educational curricula are inclusive and supportive, depending on the functional development of the child. This study has several limitations. As the initial version of this questionnaire at first was not in Chinese, the English translation may include important nuances that were not recognized. The translation should be validated in each population to accurately define the norms. The records of only 57 children included complete medical histories on their coarse facial features with broad eyebrows, hirsutism, skeletal dysplasia, degenerative joint disease, hepatosplenomegaly, macrocephaly, and hearing loss problems. To investigate the effects of these medical conditions on motor and cognitive functioning in children with MPS, larger and more comprehensive studies are needed. Besides, we did not compare the WeeFIM score in patients before and after ERT. It is necessary to assess their abilities before they have ERT and evaluate the ERT effectiveness after treatment in the future.

## 5 CONCLUSION

The WeeFIM questionnaire responses revealed that the children with MPS had better mobility than self‐care and cognition function. The results should help clinicians to identify the functional strengths and weaknesses of children with MPS and to assess their abilities before and after ERT.

## CONFLICT OF INTEREST

The authors declared that they have no conflict of interest.

## AUTHORS’ CONTRIBUTIONS

CLL and HYL performed data acquisition, statistical analyses, data interpretation, and drafted the manuscript. SPL participated in study design, data interpretation, and helped to draft the manuscript. CKC, RYT, YHH, and HCC performed biochemical analyses and revised the manuscript. WLH, FJT, PCC, DMN, YJC, MCC, TMC, JLL, CYC, and YCK were responsible for patient screening and revised the manuscript. All authors read and approved the manuscript.
